# P-1785. A nationwide survey of Antimicrobial Stewardship Infrastructure and CDC Core Elements in the Dominican Republic

**DOI:** 10.1093/ofid/ofae631.1948

**Published:** 2025-01-29

**Authors:** Elianet Castillo, Arzina Aziz Ali, Rita A Rojas-Fermin, Yeison Reyes, Claudia Blanco, Antonio M Villegas, Alfredo J Mena Lora

**Affiliations:** CEDIMAT/CEMDOE, Santo Domingo, Distrito Nacional, Dominican Republic; University of Illinois at Chicago, Chicago, Illinois; Hospital General de la Plaza de la Salud, Santo Domingo, Distrito Nacional, Dominican Republic; Hospital General de la Plaza de la Salud, Santo Domingo, Distrito Nacional, Dominican Republic; SDI, Santo Domingo, Distrito Nacional, Dominican Republic; CEDIMAT, Santo Domingo, Distrito Nacional, Dominican Republic; University of Illinois Chicago, Chicago, Illinois

## Abstract

**Background:**

Antimicrobial resistance (AMR) poses a critical global health threat, escalating the need for widespread implementation of antimicrobial stewardship programs (ASPs) to monitor and promote appropriate use of antimicrobials. Despite the growing concern for AMR, particularly in low and middle-income countries, data on the implementation of ASPs in these regions remain scarce. This study aims to describe the presence and practices of ASPs in the Dominican Republic (DR).

Figure 1

**Figure:**
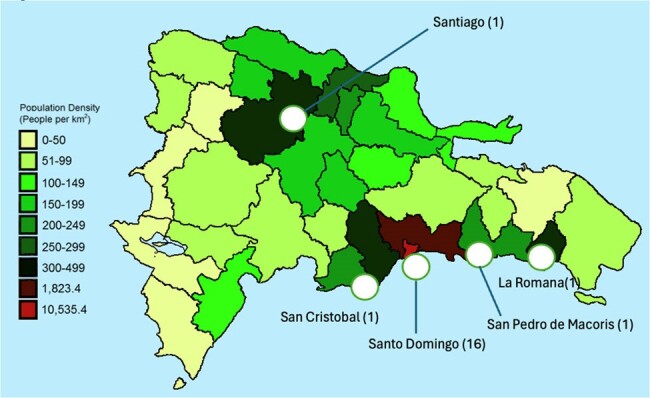

Population density of the Dominican Republic and geographic distrubution of hospitals surveyed

**Methods:**

We performed an anonymous survey of infectious diseases (ID) specialists in acute care hospitals in the DR. A survey based on the CDC Infection Control Assessment and Response (ICAR) tool for antimicrobial stewardship was distributed via professional society listervs between March 4 and April 24, 2024. Data was tabulated and descriptive statistics performed.

Figure 2

**Figure:**
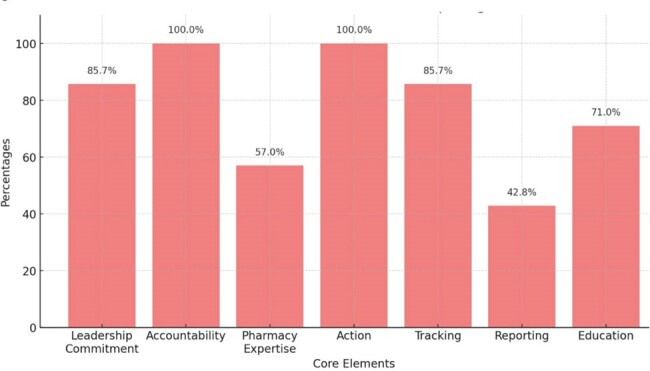

CDC Antimicrobial Stewardship Core Elements met by hospitals in the DR

**Results:**

A total of 20 hospitals completed our survey, of which 16 were from Santo Domingo and 1 from La Romana, San Cristobal, San Pedro, and Santiago (Figure 1). Eighteen were tertiary centers. ASP was absent in 50% (10), present in 35% (7), and in the process of development in 15% (3). Compliance with CDC core elements for ASP varied, with accountability and action present in all hospitals with ASP, leadership commitment and tracking both at 85.7%, and education at 71.0% (Figure 2). Pharmacy expertise and reporting were 57.0% and 42.8% respectively. The most frequently adopted ASP interventions were 'Documentation of indication' and 'Center Specific Treatment guidelines', both present at 71% (5) of hospitals. PAF and 'Pre-authorization' were also common, each at 57% (4/7) (Figure 3). The less frequent interventions included 'Antibiotic timeout' and 'Assessing Penicillin allergy', both at 14% (1/7), and 'Review of outpatient parenteral antibiotic therapy' at 29% (2/7).

Figure 3

**Figure:**
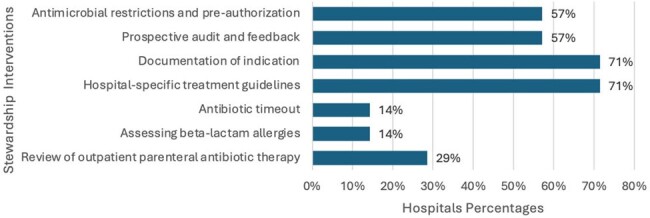

Antimicrobial steewardship interventions by hospitals in the DR

**Conclusion:**

Our survey provides a snapshot of the current state of ASP in the DR. While there has been notable progress in implementing ASPs, half of the hospitals still lack such programs, underscoring the urgent need for broader implementation. Additionally, adherence to the CDC’s core elements among existing programs varies, highlighting specific gaps and areas for improvement. It is crucial to address these deficiencies to enhance ASP effectiveness and combat the growing threat of AMR.

**Disclosures:**

**Rita A. Rojas-Fermin, MD,FIDSA**, Gilead: Advisor/Consultant|Pfizer: Advisor/Consultant|Pfizer: Honoraria

